# The Foreign Journals: Medical Statistics

**Published:** 1839-04

**Authors:** 


### MEDICAL STATISTICS.

### REVACCINATION.

### I. *Results of the Revaccination of the Prussian Army in* 1837.

THE number of troops vaccinated in this year amounted to 47,258. Of this number, distinct cicatrices of former pustules were visible in 37,299, indistinct in 6,903; and no cicatrices in 3,056. The pustules produced by the revaccination were regular in 21,308, irregular in 10,557, and no effect was produced in 15,393. The revaccination was repeated upon 12,014 of the abortive cases; successfully upon 2,243, unsuccessfully upon 9,771.

Of cases revaccinated in 1837, or at an earlier period, 14 had during this year an attack of varicella, and 7 of the varioloid disease; but there was no case of genuine smallpox.

The vaccine pustule was observed to pursue its regular course, in several cases where there were distinct marks of a former attack of smallpox; several cases of recurrent smallpox were likewise observed, and among others that of a soldier of the 40th regiment, whom, on account of the very distinct marks of the former attack, it had been thought unnecessary to revaccinate. A few cases likewise occurred, in which the vaccine pustule pursued its course simultaneously with that of other variolous eruptions.

The proportion of successful revaccinations has, as in former years, continued to increase. In 1833 the proportion was 31 per cent.; in 1834, 37; in 1835, 39; in 1836, 43; and in 1837, 45 per cent.

The total number of variolous diseases in the army during this year amounted to 94; viz. 46 varicella, 40 varioloid, and 8 genuine smallpox. The patients were chiefly recruits, and were attacked before an opportunity had offered for vaccinating them. Three fatal cases only occurred, and in two of these neither the cicatrix of the vaccine pustule nor that of natural smallpox could be recognized.

The beneficial effects of the revaccination will appear from the following data. In 1834, the number of cases of variolous eruptions amounted to 619, and the deaths to 38. In 1835, the number had diminished to 259, and the deaths to 5. In 1836, the total number was 130, and the deaths 9; and in 1837, there were only 94 cases, and 3 deaths. To understand fully the value of these figures, it must be borne in mind that during these years smallpox appeared rather to be upon the increase than decrease among the inhabitants of the country generally.*

*Medicinische Zeitung.* No. 27, 1838.

### II. *Discussions in the French Academy.*

THE majority of the French Academy of Medicine disapprove of revaccination. M. Brischet, in his report to the "Institute," places the utility of revaccination

* The results of the revaccination of 1836 are given in our Ninth Number, p. 210.

upon the affirmation of the two following propositions: viz. 1, the degeneration of the vaccine virus; 2, the failure, after a time, of the security afforded by it. Upon these two propositions M. Rochoux remarks:

"The hypothesis of the degeneration of the vaccine virus has found a recent defender in M. Guersent, and since it is continually reproduced, it should as incessantly be refuted. I am thus constrained to repeat, with respect to the first proposition,—the degeneration of the vaccine virus,—that every contagious virus is a production analogous, in some respects, to an organized being; especially in its susceptibility of indefinite reproduction, with all its specific characters. Such vitality has been manifested by the syphilitic virus for three centuries, and by the smallpox virus for upwards of twelve. The vaccine virus is governed by the same law. It may be stated, in consequence of its weekly propagation, since its first employment, to have passed through upwards of 2000 generations; and surely, had it been susceptible of diminution, it would long since have deposited all its power, though it should have lost it only by infinitesimal, homœopathic portions. I cannot, therefore, hesitate to consider that the hypothesis of the degeneration of the vaccine virus is completely disproved.

"The disproval, however, of the first proposition will not afford grounds for a satisfactory security, if the second be not disproved likewise; if, as Heim thinks, time would diminish, in the vaccinated, the power of resistance to the influence of smallpox. The number of subjects revaccinated with success in Germany has been brought forward to prove the temporary nature of the security of this operation.

| | | | | |
|---|---|---|---|---|
| Rocche mentions that . . | 37 | revaccinations took | place out of | 100 |
| The Report to the Institute, | 20,000 | ditto | ditto | 44,000 |
| M. Bousquet . . . . | 21,308 | ditto | ditto | 47,263 |

"It should however be observed, that revaccination tried in France has been attended by results very different from that in Germany.

"Since the introduction of vaccination into France, the number and severity of smallpox epidemics have progressively diminished; and the small number of the vaccinated who have taken that disease ordinarily suffer less than those whom it attacks a second time. So that if varioloid diseases have become, in the mean time, more frequent (a very questionable matter), it is but a slight evil in the place of a terrific scourge.

"Since the epidemic at Marseilles, in 1828, there have been counted 30,000 subjects vaccinated. Out of this number 2,000, that is, $\frac{1}{15}$, had been attacked; but the greatest part of them manifested only a varioloid disease, or smallpox almost uniformly benign. The number of deaths amounted to 20. Out of 2,000 more in the same town, who had formerly had the smallpox, 20 only, i.e. $\frac{1}{100}$, were attacked the second time; but amongst them the disease was generally severe, 4 deaths occurring out of the 20. While thus, from an equal number, there have been seven times more of the vaccinated attacked, these have had three times less the number of deaths.

"With facts so satisfactory as these, we have to meet in favour of revaccination the profound and significant consideration, that if it will do no good, it can do no harm! making uselessness the principle of our treatment. At this rate, a cap and bells may scare away smallpox; for, certes, if it does no good, it will do no harm! Posito uno absurdo multa sequuntur.

"The loss of all confidence in the beneficent measure of vaccination, and the spreading abroad, in consequence, an unnecessary terror of disease, are not the only evil effects of proposing its repetition from time to time. This injudicious measure would prevent our ascertaining the precise value of the vaccine virus, since the preservative power given by the first vaccination might be attributed to the second or third; and, since there would be as little reason to trust *the second as the first,* the third as the second, instead of that tranquil confidence given by a knowledge of the permanent qualities of the virus, there must be excited a perpetual agitation and alarm at every approach of the uncontrollable and hideous disease—smallpox."

*Gazette des Hôpitaux. Octobre* 2, 1838.

*Influence of Artificial Feeding upon the Mortality of Infants.*

IT would appear that the establishment of beneficent institutions in France, for the reception of illegitimate and deserted children, has not only tended to encourage profligacy, licentiousness, and cruelty, but has at length "fructified," after its own kind, in the production of still greater enormities. Such an evil tree could not but bring forth evil fruit

It was soon found in the Institutions alluded to, that the compassionate regulations of the "tours d'arrondissement," by which the wretched infants were sometimes enabled to obtain their natural food, and wholesome exposure to the air, so greatly increased the living burdens of the charity, that this mercy was withdrawn. And now the public are cooly informed of the statistical difference between natural and artificial feeding upon the mortality of infants; as if this artificial feeding had no cause in the moral wrong done to the infants by the prohibition of the only salutary course which had hitherto been open to them.

M. l'Abbé Guillard, chaplain to the General Hospital of Tours, states, in the hospital of X (which he deems it inconvenient to name), not a single infant has been suckled; all those who are received are nourished by hand, through a sucking bottle. In this hospital, a very exact account has shown that last year, out of 244 children, 197 had died at the end of the year! Of these, 116 lived only from a day to a month. Out of 127 infants in 1834, there remained only 29 at the end of the year. On the 1st of January, 1835, out of 362 infants, there remained alive only 127 at the expiration of twelve months.

The suppression of the "tours" has shown, also, in other hospitals the murderous effect of this probibition. Thus in the Hospital de Poitiers, where the habitual proportion of deaths, in the first month of life had been 12 to 100, have been now increased in that month above 35 in 100. In the Hospice de Loudun, 2 only out of 11 remained alive at the end of the year, and 4 out of 9 received in the first six months of 1835. These had been all artificially fed, and prohibited from being taken out.

At Moulins, in the first months of 1835, 128 were admitted, and 100 of these had died. *Bulletin Général de Thérapeutique.* 15*th to* 30*th Oct.* 1838.

